# Reference Values for Weight, Height, Head Circumference, and Body Mass Index in Turkish Children

**DOI:** 10.4274/jcrpe.2183

**Published:** 2015-12-03

**Authors:** Olcay Neyzi, Rüveyde Bundak, Gülbin Gökçay, Hülya Günöz, Andrzej Furman, Feyza Darendeliler, Firdevs Baş

**Affiliations:** 1 İstanbul University Istanbul Faculty of Medicine, Department of Pediatric Endocrinology, İstanbul, Turkey; 2 Boğaziçi University, Institute of Environmental Sciences, İstanbul, Turkey

**Keywords:** body mass index, height, weight, children

## Abstract

**Objective:**

This study aimed to integrate the existing updated reference standards for the growth of Turkish infants and children and to compare these values with World Health Organization (WHO) reference data, data from some European countries, and also with previous local data. Weight, height, and head circumference measurements were obtained on 2,391 boys and 2,102 girls who were regular attenders of a well child clinic and on 1,100 boys and 1,020 girls attending schools in relatively well-off districts in İstanbul. Mean number of measurements per child was 8.2±3.6 in the age group 0-5 years and 5.5±3.3 in the age group 6-18 years. All children were from well-to-do families and all were healthy. All measurements with the exception of measurements at birth, which were based on reported values, were done by trained personnel.

**Methods:**

The LMS method was used in the analyses and in the construction of the percentile charts. There is an increase in weight for age and body mass index values for age starting in prepubertal ages, indicating an increasing trend for obesity.

**Results:**

Compared to WHO reference data, weight and height values in Turkish children were slightly higher in infants and in children younger than 5 years, while they showed similarity to those reported for children from Norway and Belgium. Head circumference values, which were slightly higher than the WHO references in the first 5 years, were comparable to the data on Belgian and Norwegian children in the first 9 years of life. At older ages, Turkish children showed higher values for head circumference.

**Conclusion:**

The relatively larger head circumference values were interpreted to reflect a genetic characteristic.

**WHAT IS ALREADY KNOWN ON THIS TOPIC?**

Standards on height, weight, body mass index are available for Turkish children.

**WHAT THIS STUDY ADDS?**

This study adds standards for head circumference and values for standard deviation score of height, weight, body mass index and head circumference at 0-18 years old.

## INTRODUCTION

Assessment of growth for age based on anthropometric measurements is an important and reliable method in the monitoring of health in an individual child. Height and weight measurements are also the most frequently used indices to evaluate the nutritional state of the community.

The growth charts and reference values proposed by WHO in 2006 as an “international growth reference” are based, for the first five years of life, on the World Health Organization (WHO) Multicentre Growth Reference Study (MGRS), a population-based longitudinal study conducted on groups of breastfed infants in 6 countries. On the other hand, the reference values proposed for use in older children are based on the revised version of the National Center for Health Statistics (NCHS) that have been in use in the Unites States since 1977. These latter charts are known as the 2000 Centers for Disease Control (CDC) Growth Charts (1,2). The WHO references for older children were developed by the merging and smooth transition of these data (3).

Although local data from various countries on infants and young children show some differences from the WHO figures, overall, there is a striking similarity in the growth pattern of breastfed infants in the first years of life among many ethnic groups (4,5,6,7). The differences in growth among children of different populations become more obvious in the older age groups and strikingly so in the pubertal ages. Significant differences in final height have been reported among populations who are similar in socioeconomic level and even among those of similar geographic location (8,9,10).

In Turkey, the growth standards obtained from measurements of children born in the years 1950-60, also created by our team, have been in use by pediatricians and many pediatric centers in the past two decades (11). This paper aims to integrate the existing updated data on the growth of Turkish children from birth to 18 years and to serve as a source for reference data (7,12,13). All these studies were conducted on children of higher socioeconomic strata, living in İstanbul. Due to significant differences in child care among socioeconomic classes (SEC) in Turkey, which, despite its recent economic growth, is still a developing country, only children of families of high socioeconomic level who live in İstanbul, who we believe are able to provide optimal or near optimal care to their children, were included in this study. Since İstanbul, over the years, has been receiving emigrants from all over the country, the mixed ethnic structure of this metropolis can be assumed to be quite representative of Turkey.

## METHODS

The growth data in the age group 0-5 years were based on the anthropometric measurements of infants and children attending the Well Child Clinic of the University Hospital between the years 1992 and 2006. Children born at the Maternity Clinic of the University Hospital constituted the majority of the infants and children followed at the clinic. At discharge from the Maternity Clinic, each mother receives a pamphlet with information on the Well Child Clinic. The pamphlet stresses the importance of follow-up in baby and child care, and parents are invited to make an appointment at the clinic. Preterm infants born before 37 completed gestational weeks were not included in this study.

Families attending the Well Child Clinic are relatively homogeneous in socio-economic and cultural level. All families are well above the poverty line as assessed by their ability to bring their baby to a centre and pay for the service albeit modest. The parents of all subjects in this study were literate, and the majority of the mothers had at least 5 years of schooling. The majority of the fathers were high school graduates.

The routine follow-up schedule of the clinic for the first year of life starts at age 2 weeks, and includes visits at 1, 2, 3, 4, 5, 6, 9, 12, 15, and 18 months and every 6 months thereafter until 5 years of age. At each visit, the parents are asked to supply detailed information on the infant’s feeding regimen, on parent-child interaction, on the baby’s development, and on any health or other problems that may have come up since the last visit. A complete physical examination including anthropometric measurements is performed at each visit. Pediatric residents and nurses provide breastfeeding counselling. All data on the infants/children attending the Clinic are recorded in the computer.

Length/height, weight, and head circumference measurements are performed by two trained nurses. The naked weights are obtained on an electronic digital scale (Seca, 727), accurate to 5 g. A locally manufactured standard measuring board, with increments in millimeters, is used to measure supine length. After age 3 years, standing height is measured using a Leicester Height Measure (Child Growth Foundation, manufactured by Invicta Plastics Roadby, Leicester, UK). Head circumference is measured with a narrow non-stretch tape placed in the horizontal plane encompassing the midpoint of the forehead between the eyebrows and hairline and the occipital prominence.

The study sample consisted of 2,391 boys and 2,102 girls aged between 15 days and 60 months of age. The data set included a total of 19,523 measurements in boys and 16,807 in girls for length/height, 19,714 measurements in boys and 17,035 in girls for weight, and 16,374 measurements in boys and 14,192 in girls for head circumference (up to age of 36 months). Body mass index (BMI) values were calculated from weight and height measurements in 19,433 boys and 16,740 girls. Mean number of measurements per child was 8.2±3.6.

The sample for children of ages 6 to 18 years consisted of 1,100 boys and 1,020 girls between 6 and 18 years of age attending primary and secondary schools located in six different districts of İstanbul city. All six schools were located in relatively well-off districts. The data were collected between the years 1989 and 2002 by biannual visits to the schools by a team consisting of one pediatrician, two trained technicians, and two physicians training in pediatrics. Using the school files, all children in one class at a time, whose birthdays were ± 3 months from the prospective date of examination, were selected as subjects to be measured at the next visit. Information on the study and on the importance of height and weight measurements was given to children in groups. Written parental consent was obtained with the help of the school administration. Children who refused to cooperate were excluded. Children of younger ages (6-10 years) constituted the subjects in the first 3-4 years of the study and over time, measurements were repeated on these same children, but other children were also taken into the study to provide adequate numbers for the older age groups. Thus, the total sample consists of a mixture of children followed longitudinally over different periods of time. Chronological age was computed from the birth date reported by the child and verified by the school files. If these two sources disagreed, the child was not included in the study. Chronic or debilitating disease, assessed by history and a brief physical examination, was also a reason for exclusion.

Heights were measured in standing position with bare feet, using a portable measuring device (the Leicester height measure, Invicta Plastics, Ltd, UK). A portable scale sensitive to 0.1 kg was used for weight measurements, which were conducted with the children in their minimal underclothes. Measurement of head circumference was done as described above for younger children. All measurements were performed by the same two trained technicians. Height and head circumference measurements were repeated twice and the mean value was calculated.

After all data were collected, the subjects were allocated to SEC by the same criteria used in previous studies (11,12). This arbitrary classification is based on the education level of both parents and the occupation of the fathers. Since no significant differences were noted in height and weight values between SEC classes 1 and 2, children falling into both classes (SEC 1 and 2) were included in this present analysis. Dates of birth of the children ranged between 1974 and 1989.

The data set for children of ages 6 to 18 included a total 6,007 height measurements for boys and 5,657 for girls, 6,008 weight measurements for boys and 5,647 for girls, and 5,972 head circumference measurements for boys and 5,628 for girls. Mean number of measurement per child was 5.5±3.3. With the exception of age groups 6, 17.5, and 18 years, each half age group included measurements over 100 subjects.

The final data presented in the Tables were obtained by the merging and smooth transition of the anthropometric results of younger children with those of children older than six years.

The data were analyzed cross-sectionally. The LMS method developed by Cole in 1988 was used as the statistical technique for reference construction (14,15). This method of analysis is based on the assumption that data can be normalized by means of a power transformation, to stretch one tail of the distribution and shrink the other, thereby removing any skewness. The optimal power of a Box-Cox transformation, which obtains approximate normality, is calculated for each of a series of age groups and the trend is summarized by a curve (L). Trends in the mean (M) and coefficient of variation (S) are similarly computed. In our study, the curves were fitted as cubic splines by non-linear regression, using penalized likelihood; the extent of smoothing was controlled by equivalent degrees of freedom (16). Fitting and smoothing were done with the program LMS v. 5.1. The required centiles (C) were calculated using the following equation: C=M (1+LSZCX) 1/L, where ZCX is the normal equivalent deviate corresponding to the centile. The z score of an individual measurement was computed as Z=[(measurement/M)L-1]/(LS), where L, M, and S are the parameters (the power transformation, median, and coefficient of variation, respectively, from the Box-Cox transformation).

## RESULTS

In our sample representing the first 5 years of life, the percentage of exclusively breastfed infants was 62% at age 4 months and 26.6% at 6 months. An important fraction (62.5%) of the infants were still being breastfed, with supplementation, at 1 year of age. The percentage of children with a birthweight under 2500 g was 6.8%.

Percentile values for weight, length/height, BMI, and head circumference for Turkish boys and girls from birth to 18 years are presented in Tables 1, 2, 3, 4. LMS values and values for Z scores (standard deviation scores) are presented in Tables 5, 6, 7, 8.

## DISCUSSION

The design of the studies summarized in this paper largely conforms to the criteria suggested by Waterlow and adopted by WHO, requiring that the reference population be well-nourished, the sampling procedure clearly defined and reproducible, the sample of adequate size, the measurements relevant and of good quality, and the data adequately treated. WHO also recommends that measurements be taken at one month intervals in early infancy (17,18,19,20). In view of the fact that in Turkey, a country where large differences in economic means and lifestyle still exist among SEC, a nationally representative sample would be likely to include many children whose health has been compromised by unfavorable economic, educational, and environmental conditions and not reflect the growth potential of the population, the study population is a selective one and consists of children of well to do families. Thus, our approach to the construction of references is similar to that of WHO, “prescriptive” rather than “descriptive”, since these references will serve as a tool for the diagnosis of inappropriate growth (21).

For the interpretation of our findings, we compared the results both with WHO reference values and with reference values for Turkish children reported approximately 30 years ago. Due to lack of data on head circumference in older age groups in the WHO data, data on Norwegian and Belgian children were also included in these comparisons (Tables 9, 10) (1,11,22,23).

Up to age 5 years, weight and length/height values of the infants and children in our study were slightly higher than the WHO growth standards and similar to those reported for Norwegian and Belgian children. As was the case in the WHO sample, the proportion of exclusive breastfeeding was high in our sample and the sample consisted of Turkish children of a relatively homogenous socioeconomic group. We attribute the differences in weight and length/height observed between the WHO sample and our subjects to the quality of health care offered in the first year of life. The infants and young children in our sample were followed by frequent visits to the clinic and detailed recommendations on health care were given at each visit to all parents. Weight and length/height values in our sample were similar to those reported from Norway and Belgium up to age 9 years. Starting at this age, the Turkish sample had lower height values and slightly higher values in weight. Both weight and length/height values at all ages were higher than the Turkish reference values reported 30 years previously, indicating an ongoing positive secular trend, possibly related to improved living conditions and lifestyle, even in the higher strata of the population.

As reported previously, starting at ages 10-11 years, the children in our sample had higher values both for weight for age and BMI as compared to the WHO standards and as also compared to data reported from a number of European countries (13). These results indicate that, similar to other countries, the Turkish population is also threatened by an epidemic of obesity starting at young ages. Indeed there are a number of recent studies on prevalence of obesity in Turkish children (24), but we thought they were outside the scope of this paper and did not include them in the discussion. As is well known, this same situation exists in the US and for this reason, the reference weight data presented in the 2000 CDC growth curves and reflected in the WHO growth reference curves are based on the 1977 NCHS figures (25).

Head size appears to be greater in Turkish children in the first 5 years of life as compared to WHO figures (1). Data on head size in children older than five years are scarce. As shown in Tables 9 and 10, head circumference values were similar to those reported for Norwegian and for Belgian children up to age 12 years in boys and age 10 years in girls (22,23). Head circumference values in Turkish children were consistently higher than the Norwegian and Belgian samples starting at these ages and continuing up to adulthood. Based on these findings which indicate that the difference in head size starts at pubertal ages, it is possible to speculate that, similar to growth in height, velocity in head growth may also show a peak with onset of puberty. As reported by others, we also believe that head size is a genetically defined population trait (26). A positive association between head circumference and body size has also been reported, a finding which is in need of further investigation (27).

In conclusion, we hope and believe that this paper, in which we have attempted to bring together data on the growth of Turkish children, will prove to be useful to pediatric endocrinologists and pediatricians who work in or outside of Turkey in their clinical work.

## Figures and Tables

**Table 1:**
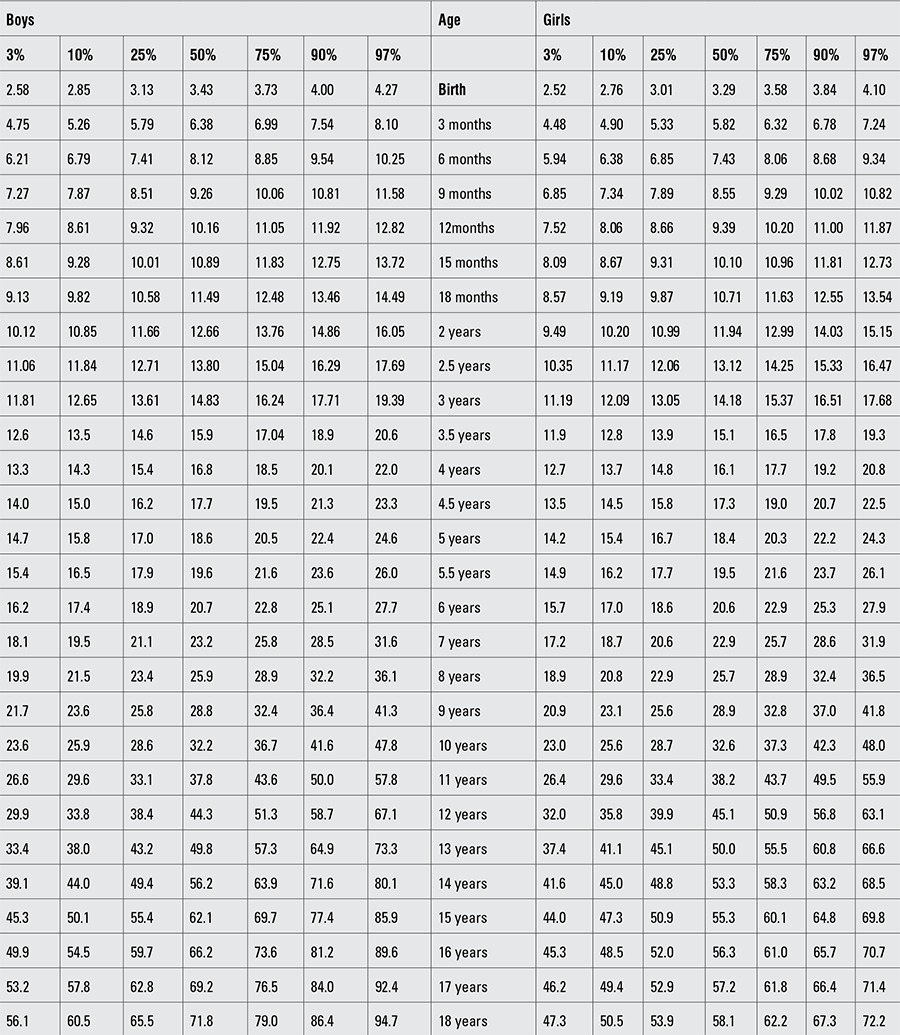
Percentile values for weight in Turkish children (kg)

**Table 10:**
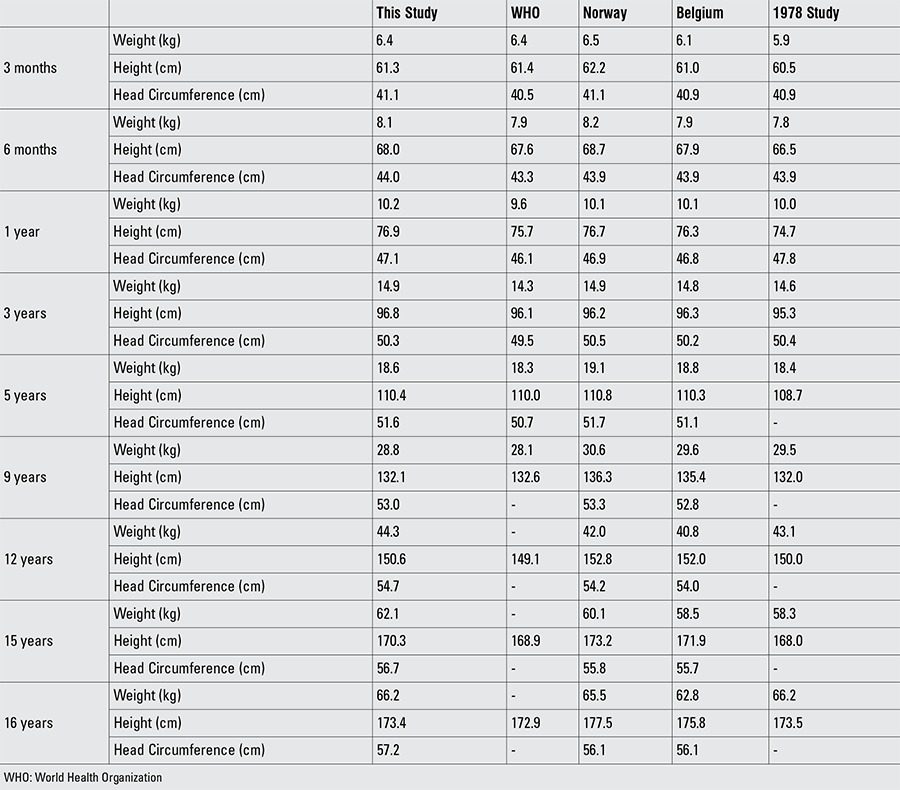
Comparison of anthropometric data in boys (mean values)

**Table 2:**
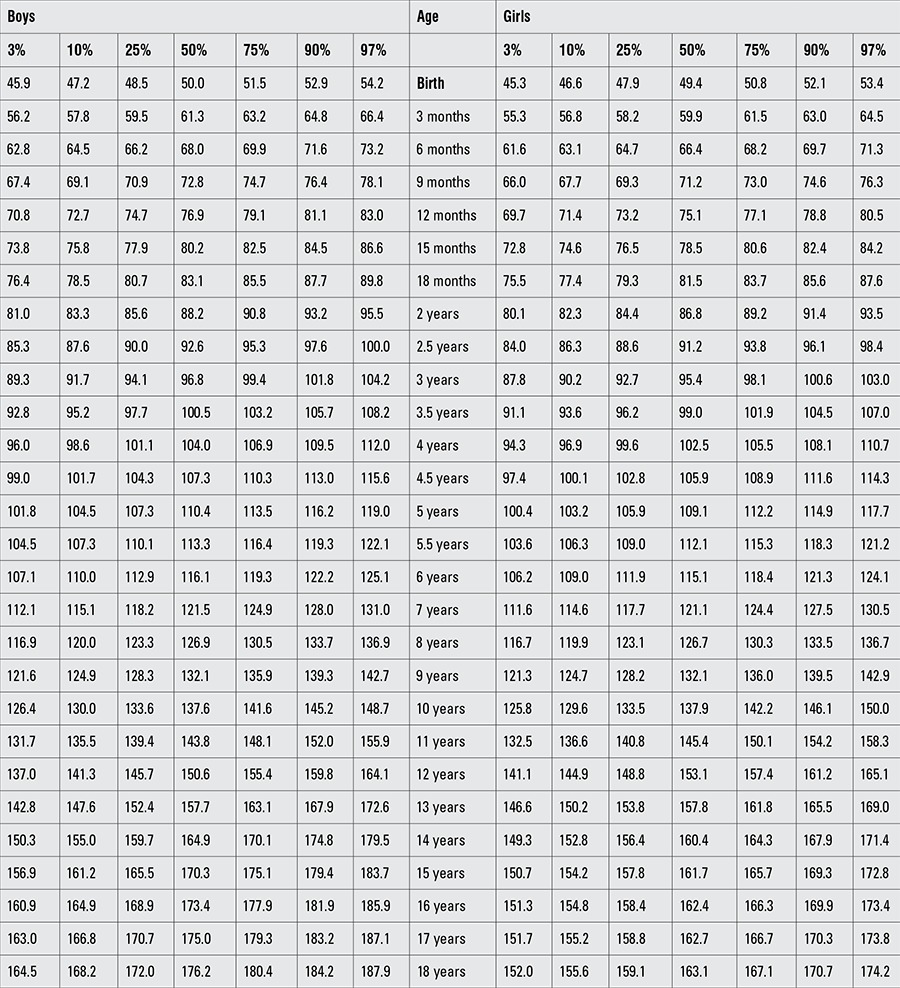
Percentile values for length/height in Turkish children (cm)

**Table 3:**
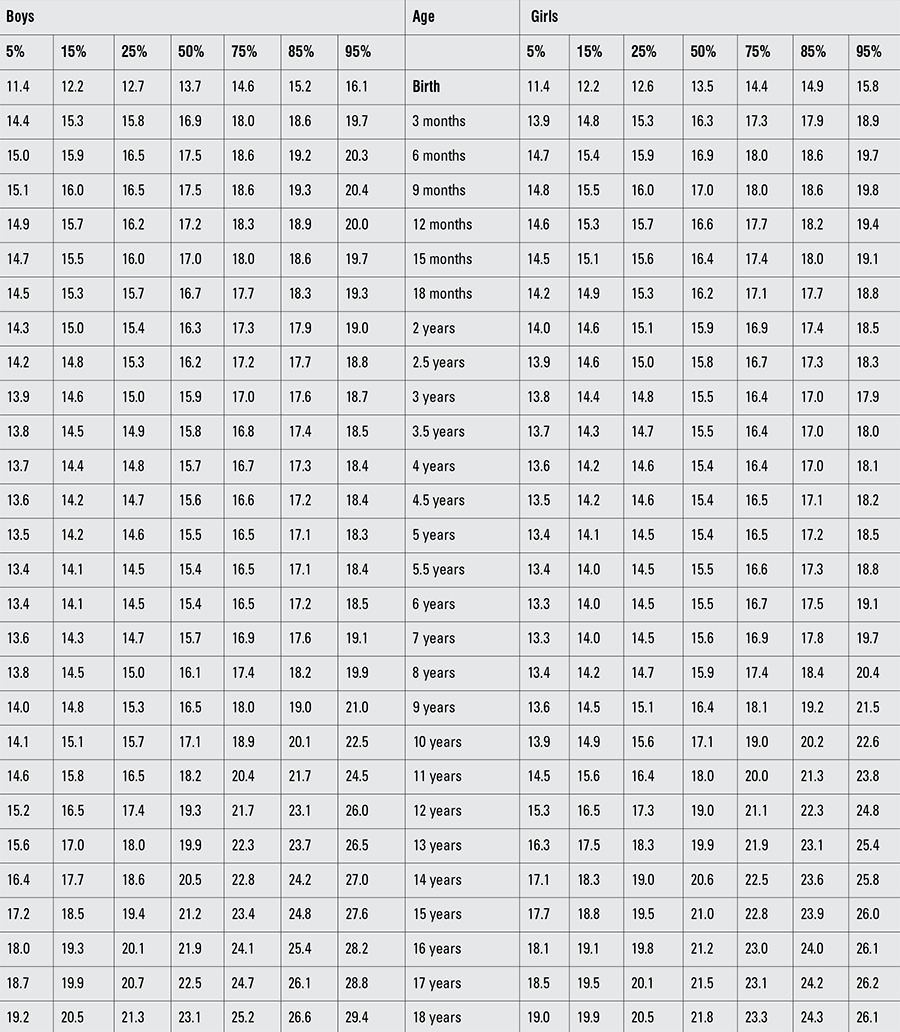
Percentile values for body mass index in Turkish children (kg/m2)

**Table 4:**
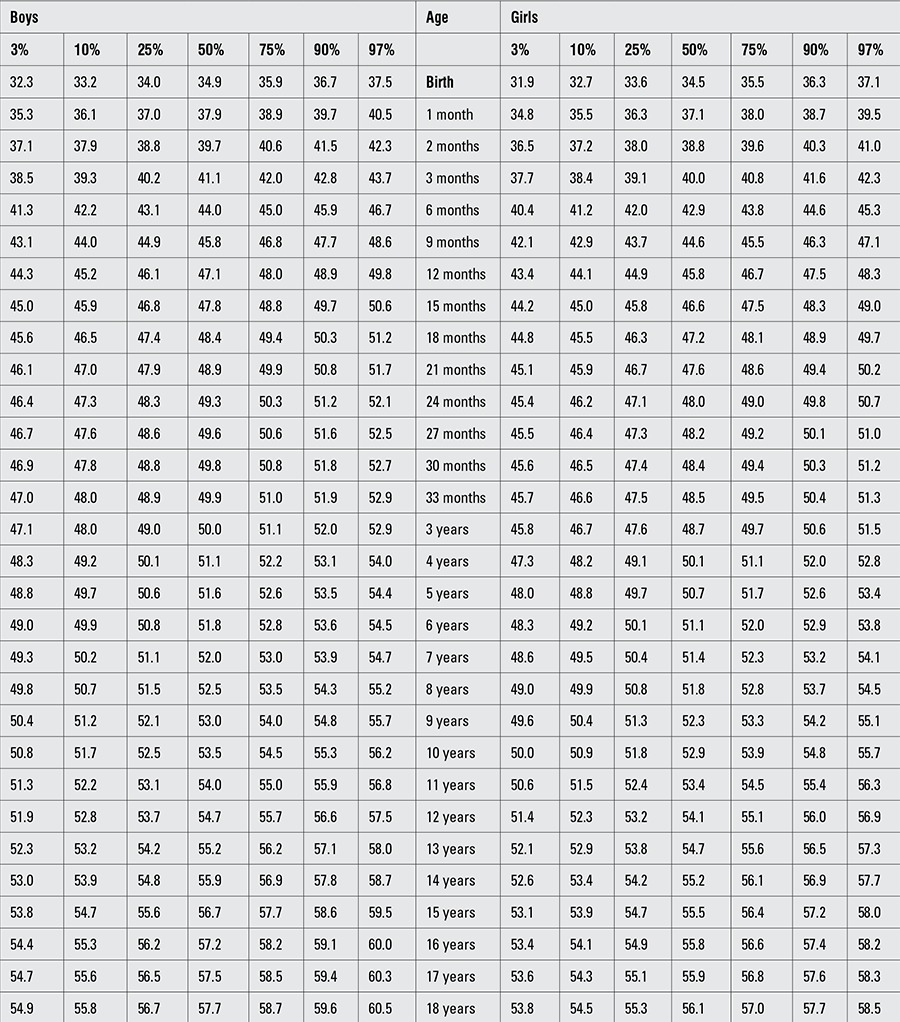
Percentile values for head circumference in Turkish children (cm)

**Table 5:**
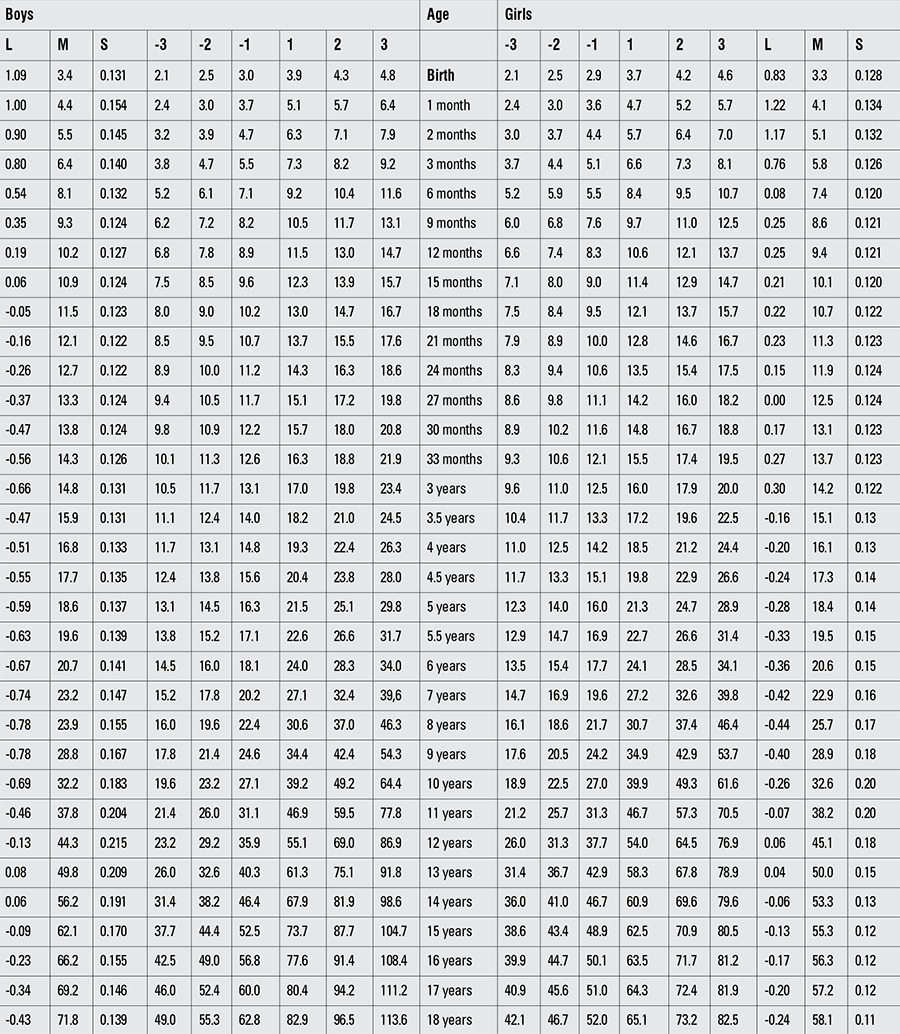
Z-score values for weight in Turkish children (kg)

**Table 6:**
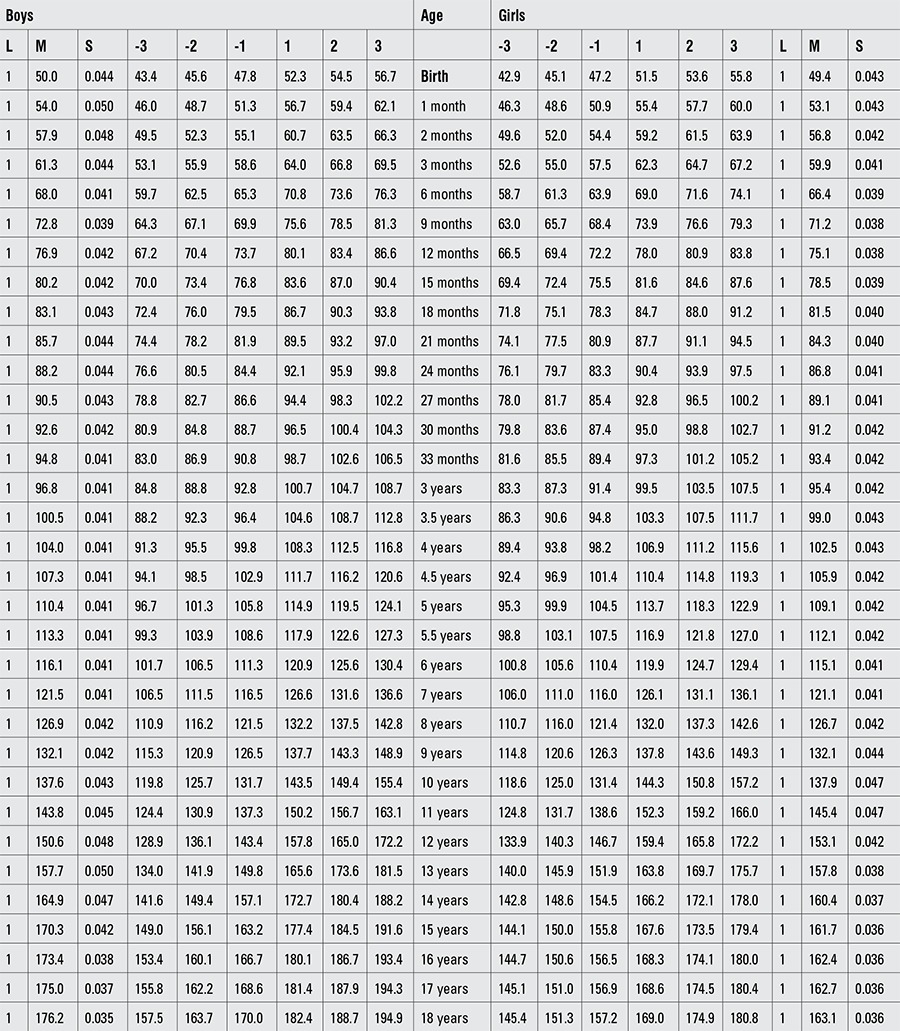
Z-score values for height in Turkish children (cm)

**Table 7:**
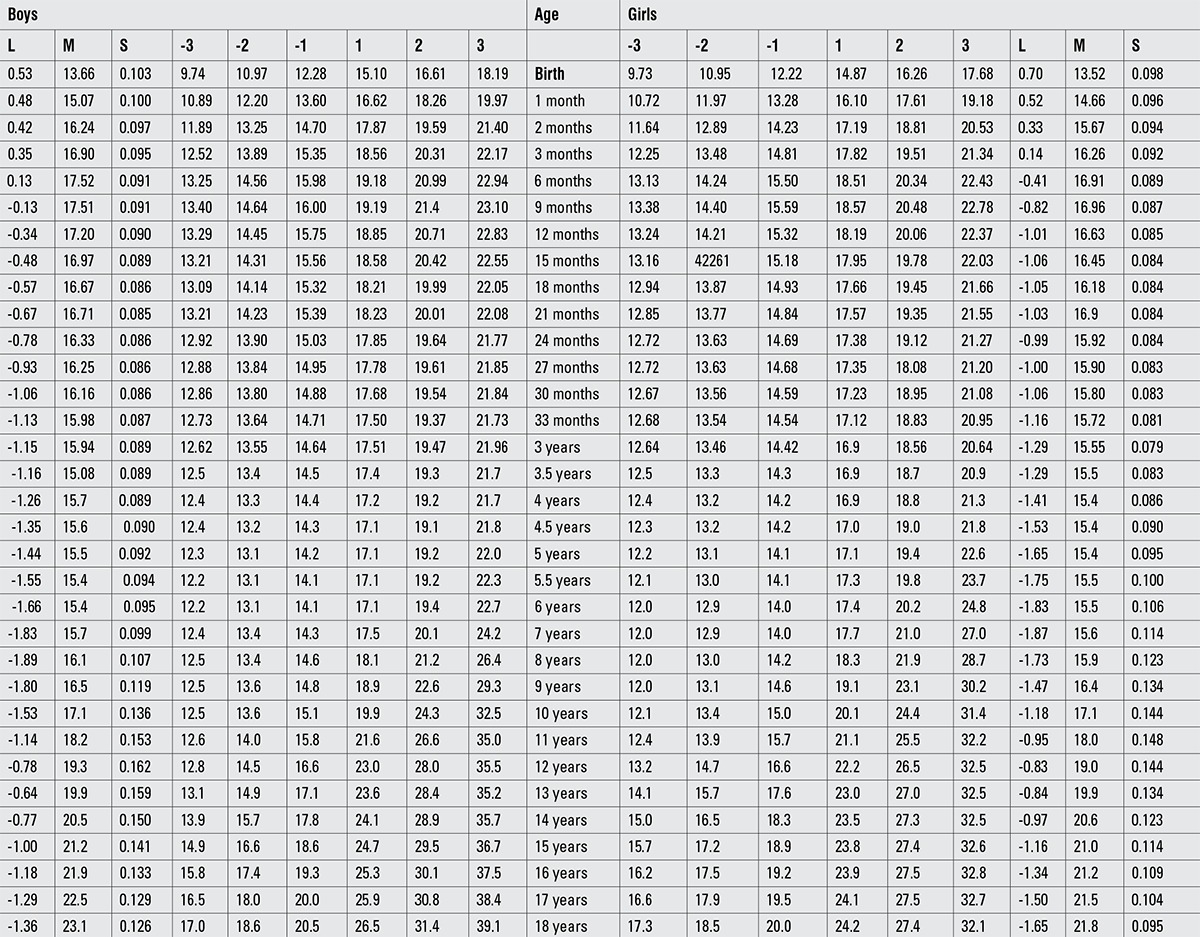
Z-score values for body mass index in Turkish children (kg/m2)

**Table 8:**
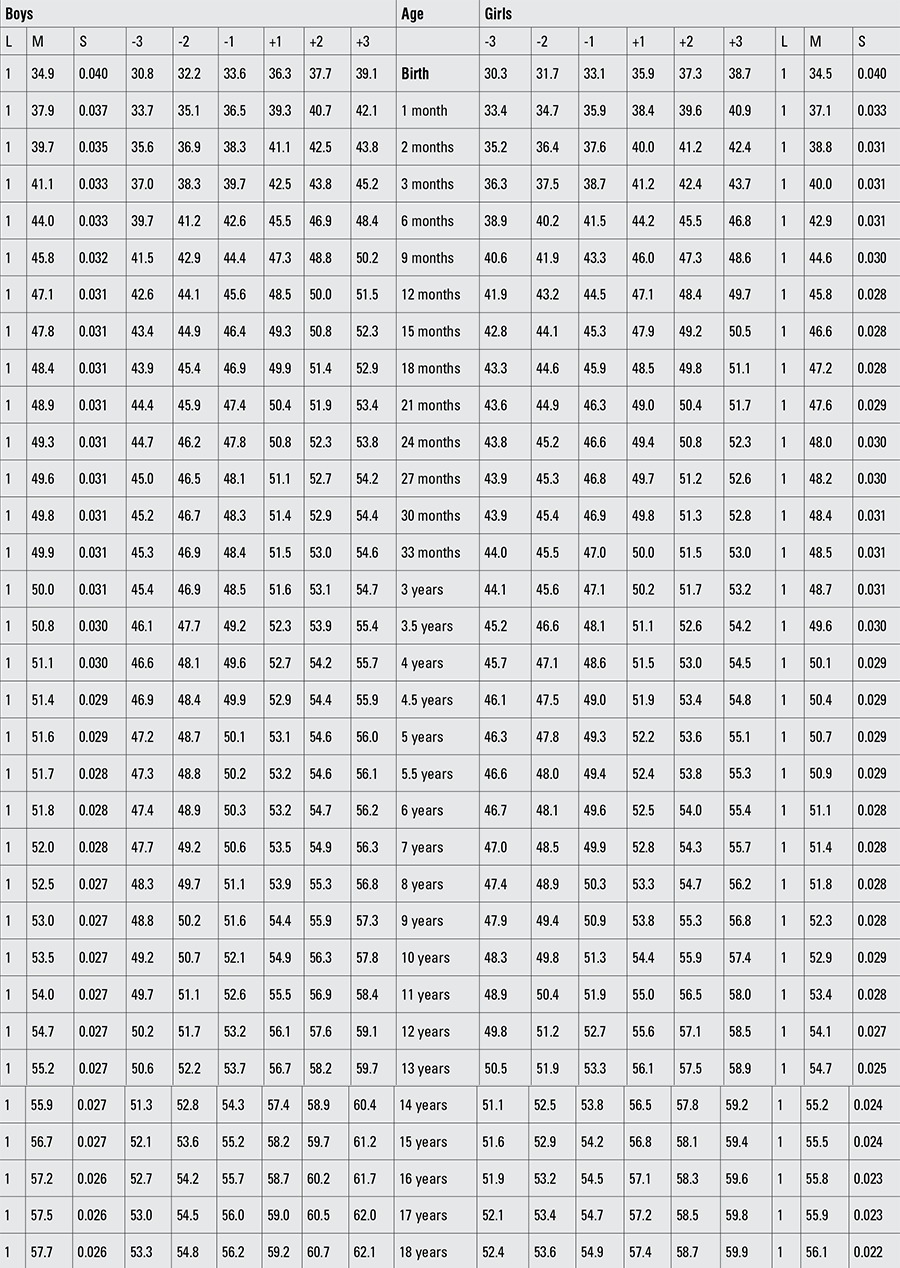
Z-score values for head circumference in Turkish children (cm)

**Table 9:**
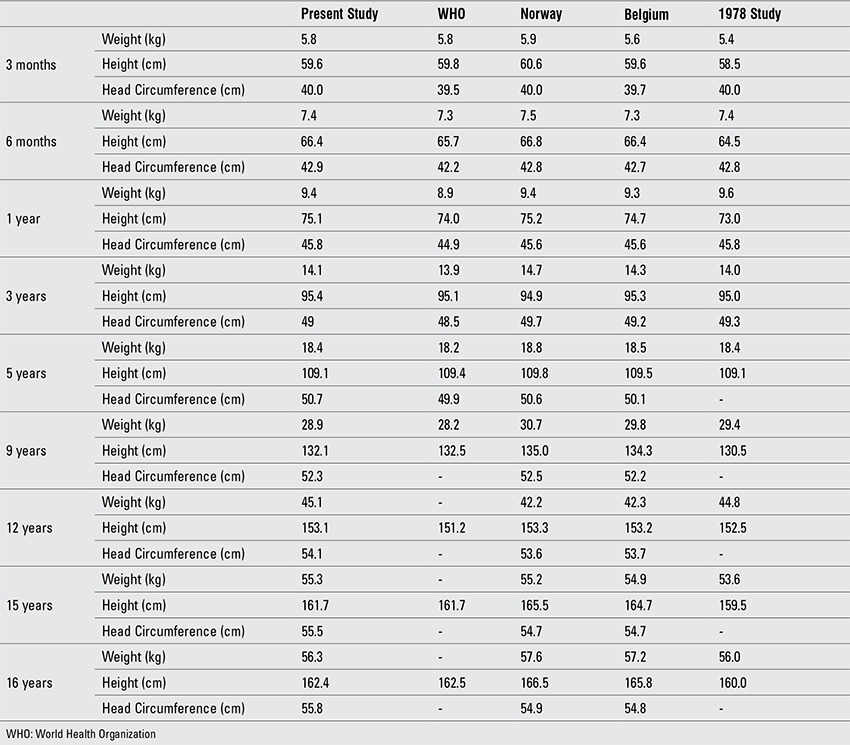
Comparison of anthropometric data in girls (mean values)
